# Shenqi Fuzheng Injection impairs bile duct ligation-induced cholestatic liver injury *in vivo*

**DOI:** 10.1042/BSR20180787

**Published:** 2019-01-25

**Authors:** Fei Cao, Peng Liu, Xianbin Zhang, Yanfen Hu, Xin Dong, Haidong Bao, Lingkai Kong, Lei Wang, Peng Gong

**Affiliations:** 1 Department of General Surgery, The Shenzhen University General Hospital; Carson International Cancer Research Centre, Shenzhen University School of Medicine, Shenzhen 518055, China; 2 Institute for Experimental Surgery, Rostock University Medical Center, Rostock 18057, Germany; 3 Institute of Cancer Stem Cell, Dalian Medical University Cancer Center, Dalian 116044, China; 4 Department of Digestive Endoscopy, The First Affiliated Hospital of Dalian Medical University, Dalian 116011, China; 5 Department of Hepatobiliary Surgery, Dalian Municipal Central Hospital Affiliated of Dalian Medical University, Dalian 116033, China; 6 Department of Hepatobiliary Surgery, The First Affiliated Hospital of Dalian Medical University, Dalian 116011, China

**Keywords:** bile duct ligation, cholestatic liver injury, inflammation, oxidative stress, Shenqi Fuzheng Injection

## Abstract

Background and aim: The aim of the present study sought to determine the protective function of Shenqi Fuzheng Injection (SFI) in cholestatic liver injury.

Methods: Cholestatic liver injury was induced in a 7-day bile duct-ligated (BDL) rat model. Rats were divided into three groups that were comprised of: (1) Sham; (2) BDL model; and (3) SFI treatment. The sham and BDL groups were treated with an appropriate volume of 0.9% sodium chloride as the vehicle, and the SFI group was administered SFI at a dose of 20 ml/kg/day, via tail vein injection.

Results: SFI significantly (all at *P*<0.01) decreased the levels of serum aspartate aminotransferase and alanine aminotransferase as compared with the BDL group, which was associated with reduced severity of inflammatory cell infiltration and hepatic damage. Moreover, SFI significantly decreased the levels of hepatic interleukin-6 (*P*<0.01), tumor necrosis factor-α (*P*=0.041), and malondialdehyde (*P*=0.026), and significantly increased the levels of total superoxide dismutase (*P*<0.01), and the GSH/GSSG ratio (*P*=0.041) in the liver. Western blot analysis showed that SFI increased PPAR-γ expression; however, SFI treatment decreased cyclooxygenase-2 (COX-2) expression and the phosphorylation of NF-κBp65.

Conclusions: These data demonstrated that SFI attenuated both inflammation and oxidative stress, and disrupted cholestatic liver injury. The involved mechanism was dependent, at least in part, on regulating PPAR-γ, COX-2, and NF-κBp65 expression.

## Introduction

Cholestasis liver injury is a condition that is usually caused by primary sclerosing cholangitis (PSC), primary biliary cholangitis (PBC), among other predisposing conditions [1]. In patients that present with this condition, pharmacological intervention remains the first clinical option to manage this disease. However, ursodeoxycholic acid (UDCA) and obeticholic acid (OCA) are currently the only two drugs that are approved by the U.S. Food and Drug Administration (FDA) to treat primary cholestasis of the liver in patients with PSC and PBC [2,3].

Although UDCA is effective in reducing serum liver enzymes, it has failed to improve liver function by histopathological examination [4]. Moreover, there are currently no clinical trials that have yet demonstrated the benefit of prescribing UDCA and OCA, either when used in combination therapy or as monotherapy, in the setting of cholestasis liver injury that is seen in PBC [2]. Thus, exploration of the potential for novel pharmacological intervention against cholestatic liver injury is urgently warranted.

Shenqi Fuzheng Injection (SFI) is a Traditional Chinese Medicine, in which the major active ingredients are found in *Radix Codonopsis* and *Radix Astragali* [5]. SFI is approved by the Food and Drug Administration of China with the intent of interfering with the side-effects that are commonly seen when administering chemotherapy [6]. Previous studies have shown that SFI attenuated irradiation-induced brain injury (RIBI) by a mechanism that was at least partly dependent on inhibiting the NF-κB signaling pathway, and reducing the levels of pro-inflammatory cytokines (e.g., Interleukin-6 or IL-6 and tumor necrosis factor-α or TNF-α) [7]. However, as far as we are aware, prior reports are lacking in the current literature that have systematically evaluated the capacity of SFI to counter the injury commonly seen in cholestatic liver injury. Additionally, until now, the mechanism by which SFI inhibits the NF-κB signaling pathway has remained unclear.

Thus, the aims of the present study were to explore whether SFI impairs cholestatic liver injury, and to determine how SFI regulates the NF-κB signaling pathway in the setting of cholestatic liver injury.

## Materials and methods

### SFI and animal experimentation

SFI was purchased from Livzon Pharmaceutics ltd. (Zhuhai, China), and was stored at 4°C until use. Male Sprague–Dawley rats that weighed approximately 200 ± 20 g were obtained from the laboratory animal center of Dalian Medical University. These rats were equilibrated for 1 week prior to the start of the experiments and were housed in standard environmental conditions (25 ± 2°C and equivalent 12:12-h light: dark cycles). Each rat was allowed access to food and water *ad libitum*. Animal experiments were performed following the National Institutes of Health Guidelines, and were approved by the local Animal Experimentation Ethics Committee of Dalian Medical University (Approval No. AEE17027).

All animals were divided into three groups: (1) Sham treated; (2) BDL model; and (3) SFI treatment. The Sham group of rats underwent laparotomy, without bile duct ligation, following which, the abdominal wall was closed. The BDL and SFI groups of rats received a midline laparotomy of approximately 1–2 cm, following which, the common bile duct was ligated with 5-0 surgical grade sutures at two positions as previously described [8].For the Sham and BDL groups, the rats were administered 0.9% normal saline (20 ml/kg/day) for 7 days by i.v. injection. For the SFI group, the rats were administered SFI (20 ml/kg/day) for 7 days, which was also done by i.v. injection [9]. At the conclusion of week one experiments, all rats were anesthetized and euthanized with pentobarbital sodium (40 mg/kg i.p.), following which, blood samples and liver tissue were collected.

Blood samples were immediately separated by centrifugation at 4°C, at a speed of 3000 rpm for 10 min to obtain the respective serum samples, which were stored at −20°C until analyzed. For each rat, the right lobe of the liver was used for histological studies and the remaining liver tissues were stored at −80°C, which were subsequently used for biochemical, ELISA and Western blot assays.

### Alanine aminotransferase and aspartate aminotransferase

Serum alanine aminotransferase (ALT) and aspartate aminotransferase (AST) activities were assayed as two reliable and sensitive biochemical markers of liver injury. The kits for measuring ALT and AST were purchased from Nanjing Jiancheng Bioengineering Institute (ALT, code C009-2, China; AST, code C010-2, China).

### Histomorphology

The liver tissue samples were fixed in 4% paraformaldehyde and embedded in paraffin after dehydration in a graded series of ethyl alcohol. Each specimen was sliced at a thickness of 4–5 μm and prepared for staining with hematoxylin and eosin (HE staining). Each tissue sample was visualized by light microscopy (Leica, DM4000B; Germany) and evaluated at a magnification of 200×. The assessment was performed by two professional pathologists.

### Assay of IL-6 and TNF-α

Two markers of inflammation (IL-6 and TNF-α) were assayed in the present study. Similar to the assays of malondialdehyde (MDA) and total superoxide dismutase (T-SOD) (see below), the concentrations of both IL-6 and TNF-α were measured by commercial assay kits that were obtained from Elabscience Biotechnology Co., Ltd (Wuhan, China), and conducted in accordance with the manufacturer’s instructions (IL-6, code E-EL-R0015c, China; TNF-α, code E-EL-R0019c, China).

### Assay of MDA, T-SOD, total glutathione (T-GSH) and oxidized glutathione

Four indictors of oxidative stress, MDA, T-SOD, GSH and oxidized glutathione (GSSG) in the liver tissues were assayed by commercially available kits that were obtained from Nanjing Jiancheng, Bioengineering Institute (MDA, code A003-1, China; T-SOD, code A001-1, China; T-GSH, code A006-2, China; GSSG, code A061-2, China).

### Western blotting analysis

Rat liver tissues (0.02g per specimen) were homogenized and subsequently lysed with ice-cold lysis buffer. Next, the samples were collected and centrifuged at 4°C, at a speed of 10000 rpm for 10 min, following which, the supernatant was stored at −80°C until final use. Western blot assays were performed as previously described [7], and were conducted with a mouse anti-PPAR-γ antibody (Santa Cruz Biotechnology, code sc-1981; Santa Cruz, U.S.A.; diluted 1:500), a rabbit anti-phosphorylated NF-κB p65 (p-NF-κBp65) antibody (Wanlei Biotechnology, code WL02169, China; diluted 1:500), a rabbit anti-cyclooxygenase-2 (COX-2) antibody (Abcam, code AB15191, U.S.A.; diluted 1:4000), and a mouse anti-GAPDH antibody (Protech, code 10494-1-AP, U.S.A.; diluted 1:8000). Quantification of proteins was analyzed using the Bio-Rad ChemiDoc XRS^+^ imaging system (U.S.A.). In this analysis, we used a random number table to select three rats from each group. All Western blot images were measured three times.

### Statistical analysis

All data are expressed as median values (i.e., the 25th and the 75th percentiles). The significance of observed differences was evaluated by the Mann–Whitney rank-sum test and Bonferroni *post hoc* correction [10]. An α-value of *P*≤0.025 was considered statistically significant and an α-value of *P*≤0.05 was considered to indicate a statistical trend. Statistical analyses were performed using Graphpad prism software, version 5.0 (Graphpad, U.S.A.).

## Results

### SFI impairs cholestatic liver injury

**Figure 1:**
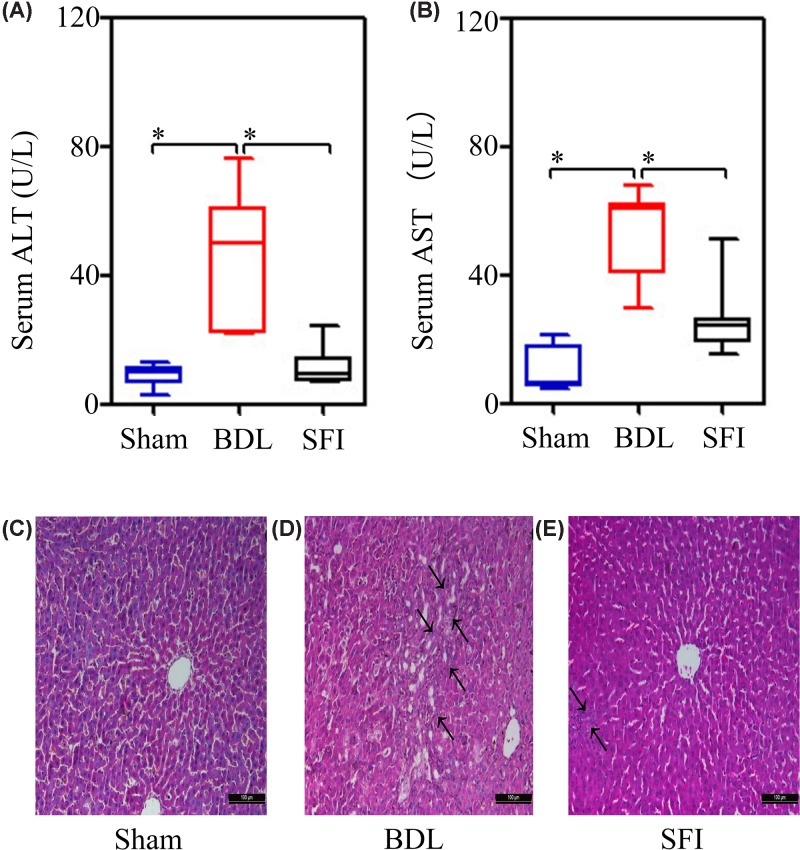
SFI impairs cholestatic liver injury Animals were treated with SFI (20ml/kg/day) in SFI group (*n*=6) or appropriate 0.9% sodium chloride in Sham (*n*=6) and bile duct ligation (BDL) group (*n*=6). Serum levels of ALT and AST were examined 7 days after operation (**A** and **B**). The arrow indicates the presence of acute cellular infiltration and hepatic damage (HE stained 200× magnifications) in Sham group (**C**), bile duct ligation (BDL) group (**D**), and SFI group (**E**).The significance of difference was evaluated using Mann–Whitney *U*-test and Bonferroni correction; * indicates significant difference, *P*≤0.025.

Compared with the Sham group, the activities of both ALT and AST were found to be significantly increased (all at *P*=0.001) in the BDL group [ALT, Sham group: 12.977 (9.511, 13.594) U/l vs. the BDL group: 45.504 (27.884, 63.176) U/l; AST, Sham group: 12.836 (6.859, 20.950) U/l vs. the BDL group: 72.631 (55.463, 85.473) U/l]. Furthermore, SFI treatment markedly decreased (*P*=0.004 and *P*=0.005, respectively) the serum levels of both ALT and AST [ALT, SFI group: 11.194 (9.500, 14.208) U/l vs. the BDL group: 45.504 (27.884, 63.176) U/l; AST, SFI group: 30.908 (25.041, 32.815) U/l vs. the BDL group: 72.631 (55.463, 85.473) U/l] (Figure 1A,B).

Histological examination showed that the hepatic tissues of the Sham group presented with normal structures with a central vein, and hepatocytes and hepatic plates being evident (Figure 1C). However, the specimens examined from the BDL group exhibited acute cellular infiltration and hepatic necrosis (Figure 1D, as shown by the arrow). Compared with the BDL group, the SFI group demonstrated that SFI attenuated the severity of inflammatory cell infiltration and hepatic damage (Figure 1E).

### SFI suppresses inflammation

**Figure 2:**
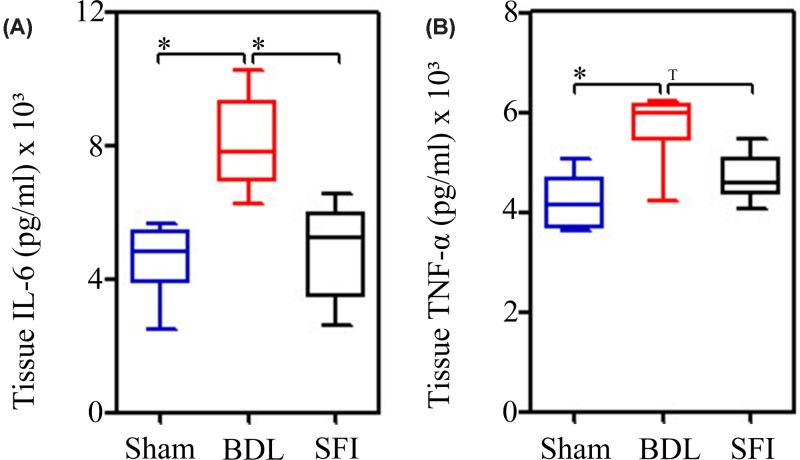
SFI suppresses inflammation in liver tissues Bile duct ligation (BDL) operation (*n*=6) significantly induced IL-6 (**A**) and TNF-α (**B**) at the 7 days, when compare with Sham operation (*n*=6). However, 20ml/kg/day SFI (*n*=6) could significantly rescue the rat underwent BDL operation (**A** and **B**). The significance of difference was evaluated using Mann–Whitney *U*-test and Bonferroni correction; * indicates significant difference, *P*≤0.025. ^T^ indicates tendency, *P*≤0.05.

As shown in Figure 2A,B, the levels of hepatic IL-6 and TNF-α were significantly increased (*P*=0.002 and *P*=0.009, respectively) in the BDL group when compared with the Sham group, [IL-6, Sham group: 4.847 × 10^3^ (4.427 × 10^3^, 5.231 × 10^3^) pg/ml vs. the BDL group: 7.818 × 10^3^ (7.276 × 10^3^, 8.726 × 10^3^) pg/ml; TNF-α, Sham group: 4.135 x 10^3^ (3.785 × 10^3^, 4.439 × 10^3^) pg/ml vs. the BDL group: 5.981 × 10^3^ (5.841 × 10^3^, 6.124 × 10^3^) pg/ml, *P*=0.009). However, SFI treatment clearly decreased the levels of hepatic IL-6 and TNF-α [IL-6, SFI group: 5.228 × 10^3^ (3.985 × 10^3^, 5.737 × 10^3^) pg/ml vs. the BDL group:7.818 × 10^3^ (7.276 × 10^3^, 8.726 × 10^3^) pg/ml, *P*=0.004; TNF-α, SFI group: 4.602 × 10^3^ (4.467 × 10^3^, 4.851 × 10^3^) pg/ml vs. the BDL group: 5.981 × 10^3^ (5.841 × 10^3^, 6.124 × 10^3^) pg/ml, at *P*=0.041].

### SFI suppresses oxidative stress

**Figure 3:**
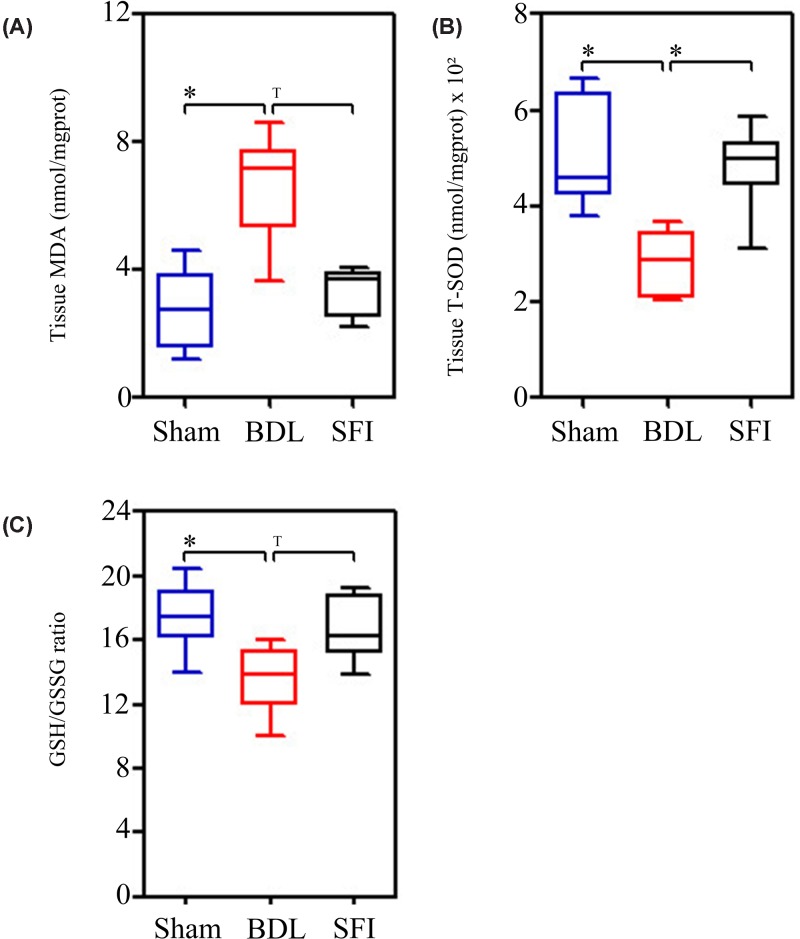
SFI suppresses oxidative stress in liver tissues Bile duct ligation (BDL) operation (*n*=6) significantly increased MDA (**A**) and decreased T-SOD (**B**) and GSH/GSSG ratio (**C**) at the 7 days, when compare with Sham operation (*n*=6). However, 20ml/kg/day SFI (*n*=6) could significantly rescue the rat underwent BDL operation (**A** and **B**). The significance of difference was evaluated using Mann–Whitney *U*-test and Bonferroni correction; * indicates significant difference, *P*≤0.025. ^T^ indicates tendency, *P*≤0.05.

As mentioned above, oftentimes, it is to be anticipated that oxidative stress amplifies the inflammatory response. Thus, we evaluated the following oxidative stress indicators: MDA, T-SOD, and the GSH/GSSG ratio in liver tissues. We found that liver MDA levels were significantly increased (*P*=0.004) in BDL rats as compared with the Sham group [Sham group: 2.746 (1.910, 3.335) nmol/mg protein vs. the BDL group: 7.170 (6.192, 7.345) nmol/mg protein]. In addition, treatment with SFI attenuated BDL-induced MDA levels [SFI group: 3.670 (2.877, 4.071) nmol/mg protein vs. the BDL group: 7.170 (6.192, 7.345) nmol/mg protein, *P*=0.026] (Figure 3A).

Contrary to the findings reported for MDA, liver tissue T-SOD was significantly decreased (*P*=0.002) in the BDL group [Sham group: 456.732 (437.622, 584.205) U/mg protein vs. the BDL group: 285.477 (226.963, 325.504) U/mg protein]. However, the levels of T-SOD were markedly (*P*=0.009) recovered in the SFI group [SFI group: 496.436 (486.964, 509.115) U/mg protein vs. the BDL group: 285.467 (226.964, 325.504) U/mg protein] (Figure 3B).

Finally, the ration of GSH/GSSG in liver tissues was significantly decreased (*P*=0.015) in the BDL group [Sham group: 17.447 (16.858, 18.314) vs. the BDL group: 13.865 (12.674, 14.962)]. However, treatment with SFI attenuated the BDL-induced GSH/GSSG ratio [SFI group: 16.264 (15.685, 18.096) vs. the BDL group: 13.865 (12.674, 14.962), *P*=0.041] (Figure 3C).

### SFI up-regulates the expression of PPAR-γ and attenuates the expression of COX-2 and the phosphorylation status of NF-κBp65

**Figure 4:**
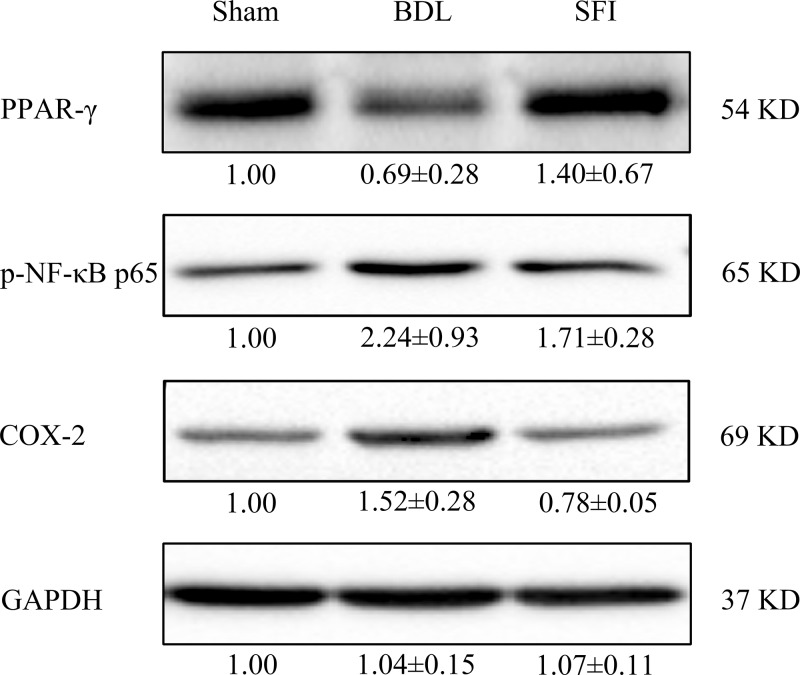
SFI up-regulates the expression of PPAR-γ and attenuates the expression of COX-2 and the phosphorylation status of NF-κB p65 At the day 7 after operation, PPAR-γ, COX-2, and NF-κB p65 were determined by Western blot. Bile duct ligation (BDL) operation (*n*=3) decreased the expression of PPAR-γ; however, it increased the level of COX-2 and p- NF-κB p65, when compared with Sham operation (*n*=3). Interestingly, 20ml/kg/day SFI (*n*=3) could restore the expression of PPAR-γ and decreased COX-2 and p- NF-κB p65.

To explore the possible mechanism underlying SFI-mediated immune suppression, we evaluated the expression of three integral proteins known to be involved in the NF-κB signaling pathway, which included PPAR-γ, COX-2, and NF-κBp65. At day seven following BDL, the levels of PPAR-γ decreased in the BDL group as compared with the Sham group; however, the expression of COX-2 and that of p-NF-κBp65 were increased. Moreover, in contrast to the BDL group, treatment with SFI (20 ml/kg/day) increased the expression of PPAR-γ, and yet by contrast, the expression levels of COX-2 and p-NF-κBp65 were decreased (Figure 4).

## Discussion

In the present study, we showed that the Traditional Chinese Medicine SFI exhibited a significantly protective effect against cholestatic liver injury in a BDL-induced rat model. Moreover, we found that SFI regulated the coordinate expression of PPAR-γ, COX-2, and NF-κB. These findings emphasize the potential use of SFI to impair cholestatic liver injury.

SFI is extracted from two traditional Chinese herbs, *Radix Codonopsis* and *Radix Astragali* [5]. Several clinical studies have previously shown that SFI markedly enhanced the immune function of patients with malignant tumors [6,11,12]. Furthermore, previous studies had found that SFI could attenuate RIBI by inhibiting neuro-inflammation [6]. Similar to RIBI, inflammation is also an important element that contributes to the initiation and progression of cholestatic liver injury [13].

Thus, in the present study, we tested the hypothesis that SFI could attenuate cholestatic liver injury. We showed that SFI significantly reduced (*P*<0.025) the serological levels of ALT and AST (Figure 1A,B), and it attenuated inflammatory cell infiltration and hepatic damage, as determined by liver histopathological analysis (Figure 1E).

However, up to now, the role contributed by various components found in *Codonopsis* and *Astragalus*, has remained largely unclear. Moreover, these components might also be found in the SFI preparation. Previous work has shown that *Codonopsis* suppressed LPS-induced lung inflammation, and dampened the expression of IL-1β, TNT-α, and NF-κB [14]. Further, another study reported that *Astragalus* reduced NF-κB DNA phosphorylation activity and decreased the levels of TNF-α, IL-6, and MPO activity in the setting of colitis [15]. In addition, Chu et al. have reported that *Codonopsis* displayed an anti-oxidative effect by decreasing MDA levels and increasing GSH-PX in a mouse model of chronic obstructive pulmonary disease [16]. Chang et al. also showed that *Astragalus* decreased intracellular ROS levels, increased the activity of SOD, and lowered the levels of MDA [17]. Therefore, since SFI was prepared from *Codonopsis* and *Astragalus*, prior studies suggested that the observed therapeutic effects of SFI might be related to the anti-inflammatory and anti-oxidative activities of both of those traditional Chinese medicinal herbs.

In the present study, we found that SFI attenuated the expression of pro-inflammatory mediators that included both TNF-α and IL-6, as well as displaying a remarkable ability to dampen MDA levels, increase SOD levels and crucially increase the GSH/GSSG ratio (Figures 2 and 3).

Zhang et al. previously demonstrated that SFI inhibited the expression of NF-κB, and played an anti-inflammatory role by inducing the NF-κB signaling pathway. However, the mechanism by which SFI is thought to inhibit the NF-κB signaling pathway remains poorly understood. Previous studies showed that PPAR-γ and COX-2 were involved in the NF-κB signaling pathway in cholestatic liver injury [18,19]. Obstructive jaundice was found to decrease hepatic PPAR-γ expression. This was an important observation since PPAR-γ activation inhibits the expression of several inflammatory response genes, including those of TNF-α and COX-2 [18]. Further, COX-2 was activated following the mobilization of NF-κB signaling in the BDL rat model, and decreased expression of both COX-2 and NF-κB, and thus attenuated inflammation in the BDL-induced rat model [20,21]. Thus, in the present study, we suggest that the protective effect of SFI might be due to inhibition of COX-2 and NF-κB expression by a mechanism that might be partly dependent on up-regulated functional expression of PPAR-γ.

**Figure 5:**
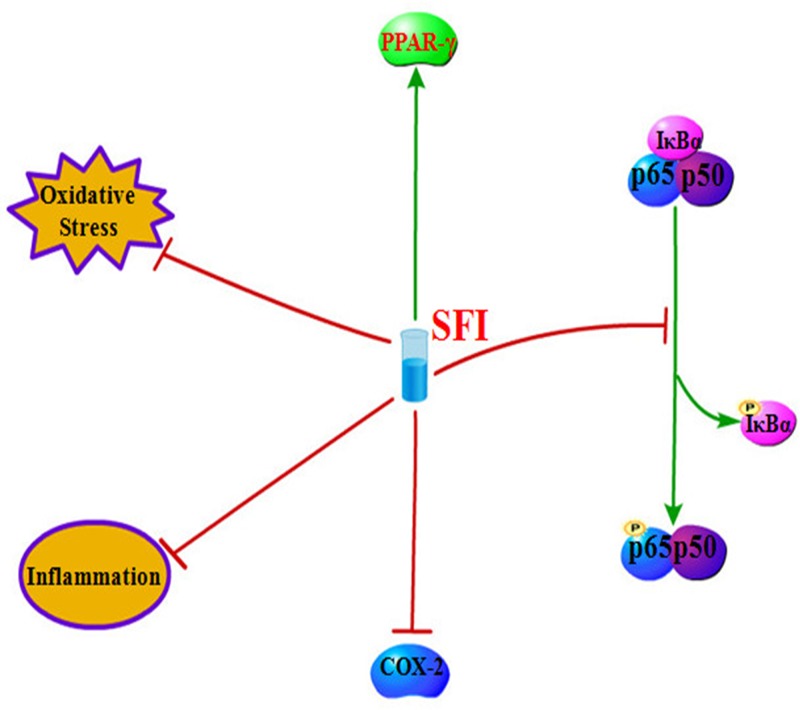
The possible mechanism of SFI to impair cholestatic liver injury in BDL-induced rats SFI attenuates the inflammation and oxidative stress to impair cholestatic liver injury. Moreover, it can increase the expression of PPAR-γ and decreases COX-2 expression. Additionally, SIF blocks the phosphorylation of NF-κB p65.

In conclusion, we have demonstrated that SFI treatment impaired inflammation and oxidative stress in cholestatic liver injury, and regulated the functional expression of PPAR-γ, COX-2, and NF-κB (Figure 5). These findings highlight the anti-inflammatory and anti-oxidative potential of SFI. We also believe that SFI promises to display clinical utility in treating patients with cholestatic liver injury.

## Abbreviations

ALT: alanine aminotransferase
AST: aspartate aminotransferase
BDL: bile duct-ligated
COX-2: cyclooxygenase-2
GSH: glutathione
GSSG: oxidized glutathione
HE: hematoxylin and eosin
OCA: obeticholic acid
PBC: primary biliary cholangitis
PSC: primary sclerosing cholangitis
SFI: Shenqi Fuzheng Injection
T-SOD: total superoxide dismutase
UDCA: ursodeoxycholic acid
